# Paraneoplastic limbic encephalitis associated with lung cancer

**DOI:** 10.1038/s41598-018-25294-y

**Published:** 2018-05-01

**Authors:** Kaini Shen, Yan Xu, Hongzhi Guan, Wei Zhong, Minjiang Chen, Jing Zhao, Longyun Li, Mengzhao Wang

**Affiliations:** 1 Department of Internal Medicine, Peking Union Medical College Hospital, Chinese Academy of Medical Sciences and Peking Union Medical College, Beijing, 100730 People’s Republic of China; 2 Department of Respiratory Medicine, Peking Union Medical College Hospital, Chinese Academy of Medical Sciences and Peking Union Medical College, Beijing, 100730 People’s Republic of China; 3 Department of Neurology, Peking Union Medical College Hospital, Chinese Academy of Medical Sciences and Peking Union Medical College, Beijing, 100730 People’s Republic of China

## Abstract

Paraneoplastic limbic encephalitis (PLE) is a rare autoimmune neurological syndrome observed in lung cancer patients. We retrospectively investigated the clinical characteristics, treatment responses, and prognoses in 16 PLE patients who were subsequently diagnosed with lung cancer. Fifteen patients initially presented with disturbance of consciousness, 13 with disorientation, and 12 with seizures. Thirteen patients had autoantibodies, including eight with gamma aminobutyric acid B receptor (GABA_B_R) antibodies and eight with Hu antibodies. PET-CT revealed lung neoplasms in 13 patients, nine of whom exhibited abnormal metabolic activity in the temporal lobe and hippocampus. Fifteen cases were confirmed as limited-stage small cell lung cancer and one as stage IV large cell neuroendocrine carcinoma. Eleven patients received immunomodulatory therapy, and four showed neurological improvement, who all had antibodies against GABA_B_R. Fifteen patients received chemotherapy, of which 14 maintained or improved their PLE status. The overall cancer response rate was 75%, and two-year overall survival was 74.7%. Our results suggest patients with GABA_B_ encephalitis might respond better to immunotherapy than the classical PLE patients with anti-Hu antibodies. Anti-cancer treatment could further improve neurological symptoms. Lung cancer patients with PLE, especially those in limited stage, might have better outcome due to earlier diagnosis and prompt anti-cancer treatment.

## Introduction

Paraneoplastic limbic encephalitis (PLE) is a rare neurological syndrome associated with cancer, and selectively affects limbic system structures, including the hippocampus, hypothalamus, and amygdala. Patients often present with cognitive impairment, personality change, short-term memory loss, and seizures^1^. PLE is an immune-mediated response associated with anti-neuronal antibodies. Classical limbic encephalitis (LE) with temporal lobe seizures is associated with onconeural antibodies directed against intracellular antigens, including anti-Hu^2^, anti-Ma2^3^, anti-amphiphysin, and anti-CRMP5^2,4^. Patients previously characterized as “seronegative” may test positive for autoantibodies to neuronal extracellular epitopes, such as the voltage-gated potassium channel (VGKC) complex, N-methyl-D-aspartate receptor (NMDAR), alpha-amino-3-hydroxy-5-methyl-4-isoxazolepropionic acid receptor (AMPAR), and gamma aminobutyric acid B receptor (GABA_B_R)^5^.

PLE is most frequently associated with lung cancer (approximately 50% of PLE cases), and 80% of these cases are small cell lung cancer (SCLC)^4^. PLE treatment options include immunotherapy and anti-cancer therapy^6^. Immunomodulators, such as steroids and intravenous immunoglobulin (IVIg), have been used to treat all types of LE with variable results. PLE associated with the classical onconeural antibodies is frequently unresponsive to immunomodulatory monotherapy. Anti-cancer therapy in combination with immunomodulation appears more effective at improving neurological symptoms than immunosuppressive treatment alone^1^. However, anti-cancer treatment is often not recommended in PLE patients, due to severe PLE-associated neurological symptoms (seizures, dementia, status epilepticus, etc.). Our understanding of PLE mechanisms has increased over the past decade, and studies have revealed that LE patients with tumors have lower survival rates than those without tumors^7^. However, few studies have focused on treatment responses and prognoses in PLE patients with lung cancer. The current study investigated clinical characteristics, autoantibody profiles, responses to treatment, and prognoses in 16 patients with LE who were later diagnosed with lung cancer.

## Results

### Patient characteristics

**Figure-1:**
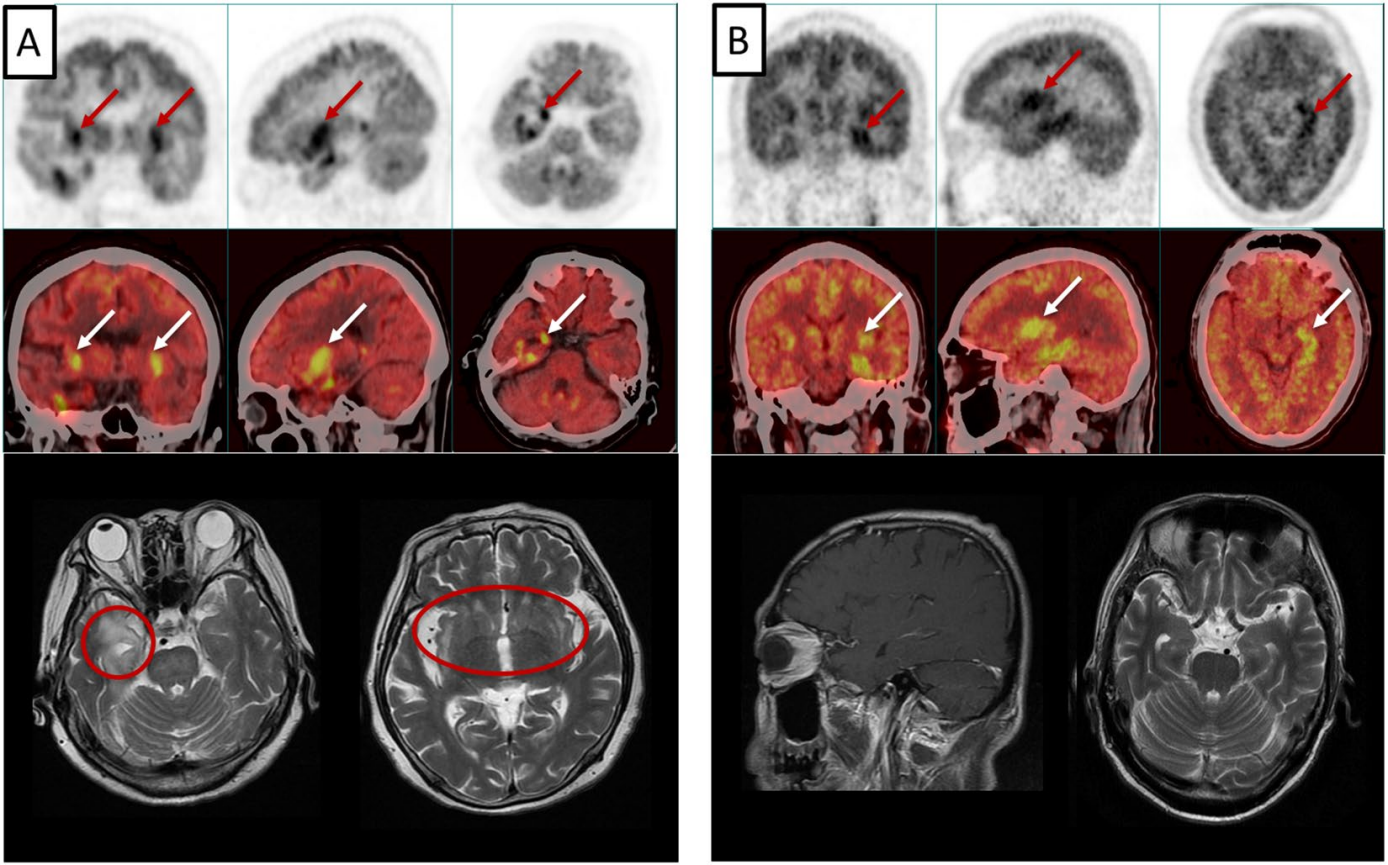
PET-CT and MRI images for two cases. Case 1: 71-year-old female (**A)** PET-CT showed enhanced metabolism in the bilateral mesial temporal lobe and insula (arrows), and MRI T2 showed high signal in the same location (circles). Case 2: 54-year-old male (**B**) PET-CT showed abnormal metabolism in the left temporal lobe (arrows), while MRI was normal.

Baseline patient characteristics are shown in Table 1. The median age at presentation was 59 (range 44–64) years with a male/female ratio of 7. Fifteen patients initially presented with disturbance of consciousness, 13 with disorientation, and 12 with seizures. Three patients had dysphagia and one had sensory polyneuropathy. LE patient antibodies, cerebrospinal fluid (CSF) analyses, electroencephalography (EEG), and imaging results are shown in Table 2. Thirteen patients were positive for autoantibodies. GABA_B_R antibodies were found in both CSF and serum samples in seven patients, and solely in serum in one patient. Ten cases had additional antibodies: eight had anti-Hu, and one each had anti-Yo, anti-amphiphysin, anti-ganglioside (GM1). The concordance between serum and CSF was greater for anti-GABA_B_R than for anti-Hu antibodies. Three different kinds of paraneoplastic antibodies were found together in two SCLC patients, reflecting a multifaceted, intraindividual immune response to multiple neuronal proteins expressed in neuroendocrine differentiated tumors. CSF cytology studies showed inflammatory changes with mildly to moderately increased lymphocytes in 8/12 cases. EEGs exhibited focal or global slow waves in 10 patients and sharp waves in two, in one case combined with epileptic activity. MRI T2-fluid attenuated inversion recovery (FLAIR) showed high signal in the bilateral mesial temporal lobes of four patients. PET-CT of nine patients confirmed abnormal metabolism in the temporal lobe, hippocampus, and cerebral cortex. Figure 1 shows MRI and PET-CT images from two cases.

**Table 1:** Baseline characteristics of LE patients with lung cancer.

Characteristics	Values
Age, years, median (range)	59 (44–71)
Sex, M/F	14/2
Smoker/non-smoker	13/3
Tobacco, pack/years, median (range)	40 (4.5–80)
Symptoms of LE, n (%)	
Consciousness disturbance	15 (93.8)
Disorientation	13 (81.3)
Generalized convulsive seizures	12 (75.0)
Memory deficits	10 (62.5)
Hallucination	3 (18.8)
Gait and stance ataxia	5 (31.3)
Disturbance in chronobiologic rhythms	5 (31.3)
Dysphagia	3 (18.8)
Sensory polyneuropathy	1 (6.3%)
Histology	
SCLC	15
LCNEC	1
Cancer stage	
Limited-stage	15
Extensive-stage	1

F, female; M, male; LCNEC, large cell neuroendocrine carcinoma; LE, limbic encephalitis; SCLC, small cell lung cancer.

**Table 2:** Antibodies, CSF analyses, EEGs, and imaging results in LE patients with lung cancer.

Examinations		Positive	Negative	Not available
Antibodies in serum	Anti-GABA_B_R in serum	8	1	7
	Anti-Hu in serum	8	6	2
	Anti-amphiphysin in serum	2	13	1
	Anti-GM1 in serum	1	15	0
	Anti-Yo in serum	1	13	2
Antibodies in CSF	Anti-GABA_B_R in CSF	7	1	8
	Anti-Hu in CSF	3	10	3
CSF	Abnormal pressure	1	12	3
	Lymphocytic pleocytosis	8	4	4
EEG		10	2	4
MRI T2/FLAIR		4	12	0
PET-CT	Head	9	4	3
	Chest	13	0	3

CSF, cerebrospinal fluid; EEG, electroencephalography; FLAIR, fluid attenuated inversion recovery; GABA_B_R, gamma aminobutyric acid B receptor; GM, ganglioside; LE, limbic encephalitis.

**Figure-2:**
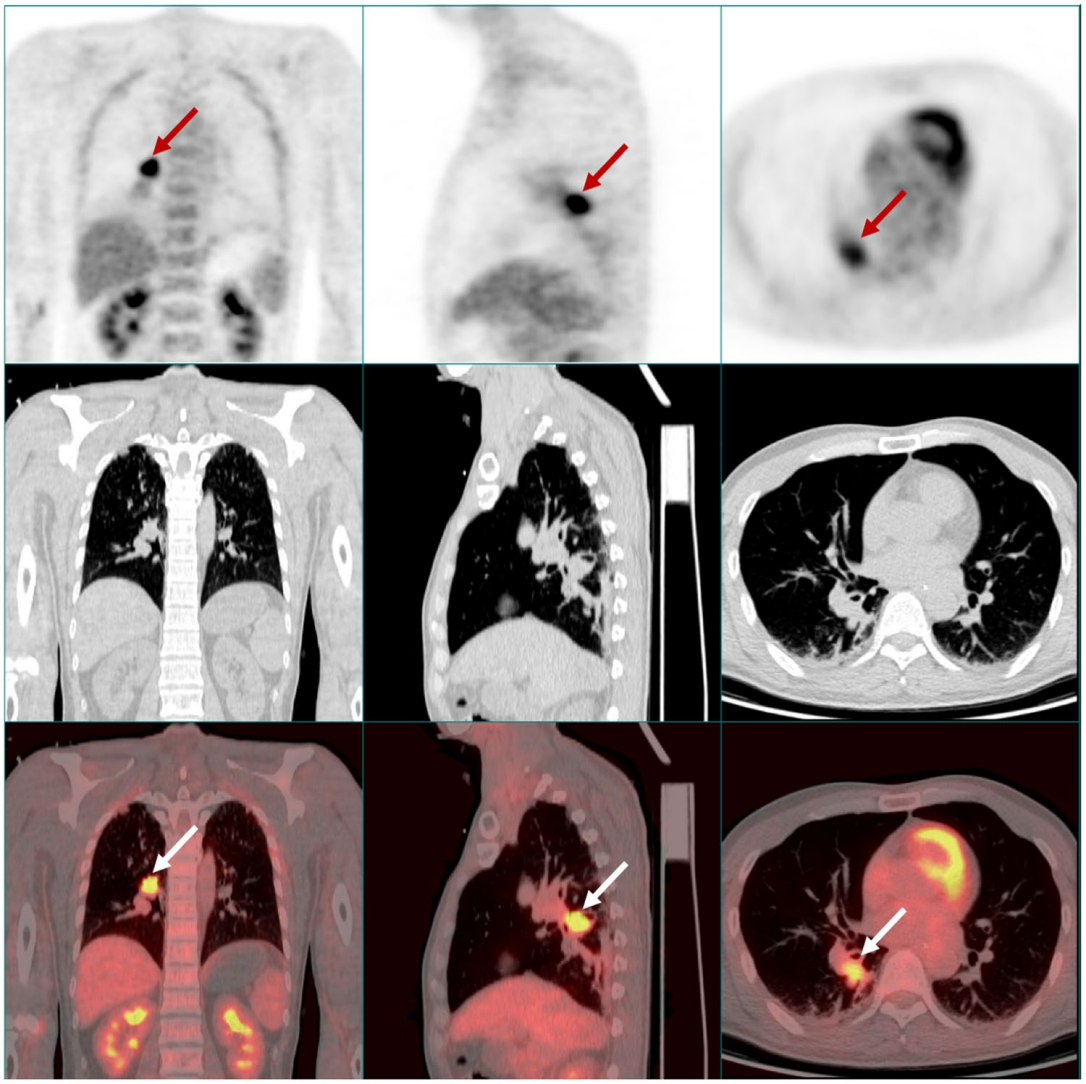
Contrast-enhanced CT and PET-CT images from a 54-year-old male patient. PET-CT showed a soft tissue mass in the inferior lobe of the right lung with abnormal metabolism (arrows), which was not evident on CT scan.

The median interval from limbic symptoms to cancer diagnosis was 66 (range, 18–234) days. After tumor assessment, such as CT of the chest and abdomen or PET-CT, lung neoplasms with or without mediastinal enlarged lymph nodes were found in all patients, 15 of whom were subsequently diagnosed with SCLC through biopsy and immunohistochemistry (Table 1). All the 15 SCLC patients were diagnosed at limited stage. One patient was diagnosed with stage IV large cell neuroendocrine carcinoma. Figure 2 shows contrast-enhanced CT and PET-CT images from one patient.

### PLE response to immunotherapy and anti-cancer treatment

Eleven patients received LE-related immunotherapy with a single agent or combination of IVIg and high-dose steroids (Table 3). Agitation and irritability improved in four patients, who became alert, with no recurrence of seizures. Some degree of memory impairment and disorientation was sustained in 2/4 patients. All patients who experienced neurological symptom improvement had antibodies against GABA_B_R. Half (50%, 4/8) of patients with anti-GABA_B_R antibodies, but none negative for these antibodies, achieved neurological symptom remission. Of the four patients who achieved remission, three received IVIg plus steroids. Limbic symptoms persisted in the other seven patients.

**Table 3:** Treatment of LE and lung cancer.

Diagnosis and treatment	Number of patients
**LE immunotherapy**	
IVIg	2
Steroids	2
IVIg + steroids	7
Untreated	5
**Lung cancer treatment**	
Chemotherapy	15
Radiotherapy	9
VATS	2
Untreated	1
**Lung cancer response**	
CR + PR	8
SD	4
Not available	3

CR, complete remission; IVIg, intravenous immunoglobulin; LE, limbic encephalitis; PR, partial remission; SD, stable disease; VATS, video-assisted thoracoscopic surgery.

**Figure-3:**
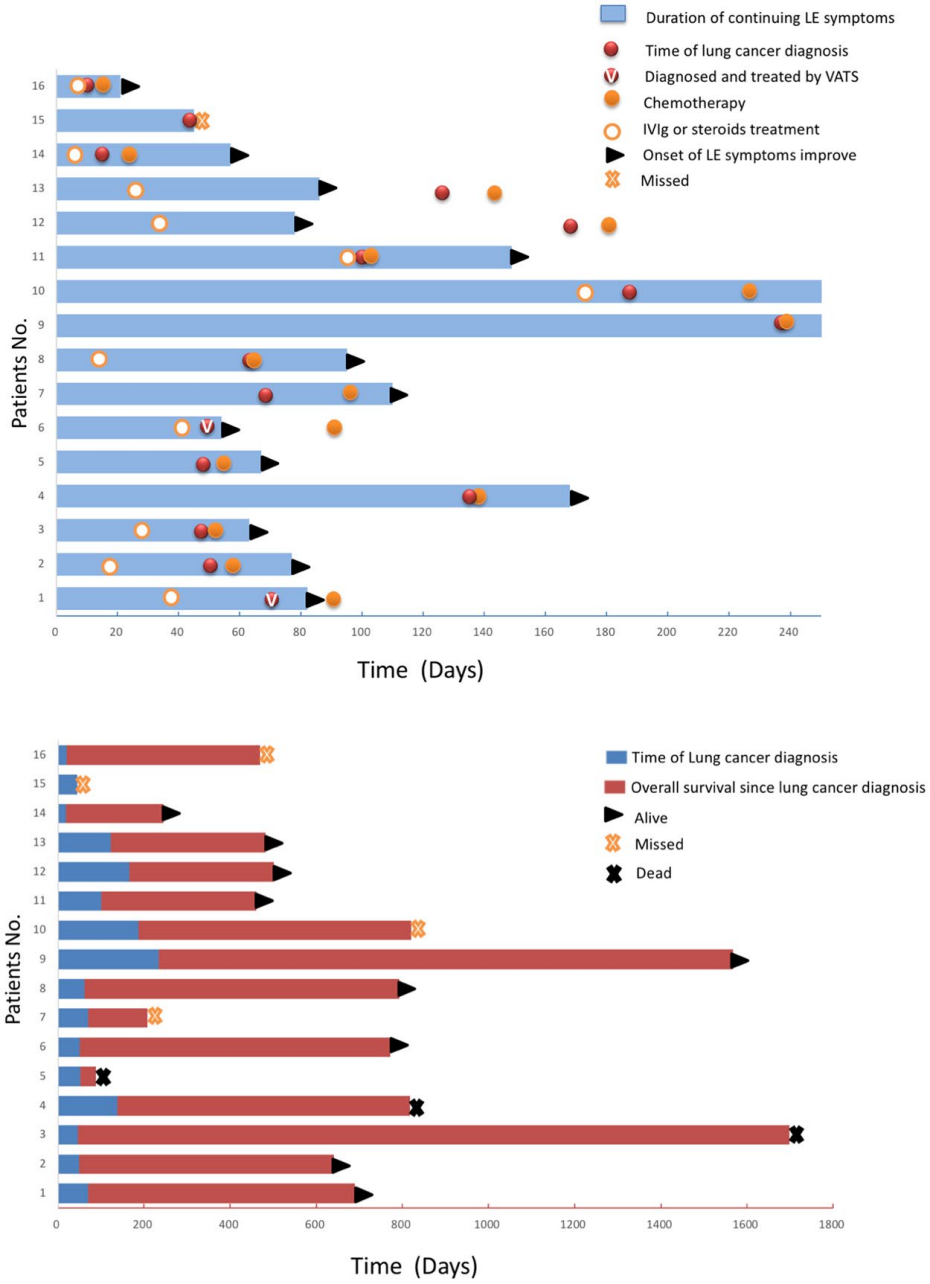
Neurological improvement and long-term survival in PLE patients with lung cancer. (**A**) Relationship between neurological symptom improvement and PLE-related treatment. (**B**) Long-term survival of PLE patients since LE symptom onset. IVIg, intravenous immunoglobulin; LE, limbic encephalitis; VATS, video-assisted thoracoscopic surgery.

Fifteen patients received platinum and etoposide-based chemotherapy after their lung cancer diagnosis. Nine of these received radiation therapy after chemotherapy and two received video-assisted thoracoscopic surgery (VATS) before chemotherapy. One refused any anti-cancer treatment (Table 3). All 11 patients treated with IVIg and/or steroids received further anti-cancer treatment. Two patients in partial remission from PLE before chemotherapy were upgraded to complete recovery and two patients in complete recovery before chemotherapy maintained their response statuses afterwards. After mediastinal lymph node resection via VATS, two patients no longer experienced seizures. Among the seven patients who did not respond to immunotherapy, four achieved complete response and three achieved partial remission of LE after anti-cancer treatment. Among the five patients who did not receive any immunotherapy for LE, four received anti-cancer treatment. Two were fully recovered after chemotherapy and limbic symptoms for one patient were partially improved. Of the 15 patients receiving anti-cancer treatment, 14 maintained or improved their LE response statuses after anti-cancer treatment. The median interval from anti-cancer treatment administration to onset of LE response was 19 days (Figure 3).

### Cancer response and long-term survival

Of the 15 patients receiving anti-cancer treatment, lung cancer response could be assessed in 12 patients after first-line treatment, with an overall response rate of 75%. Three patients achieved a complete response (CR), five a partial response (PR) and four stable disease (SD). Of the eight patients achieving as least PR, six had antibodies against GABA_B_R. Within a median follow-up duration of 17.4 (range, 1.0–55.1) months, two patients died due to tumor progression and one of chemotherapy-related infection. Two-year overall survival was 74.7% in this patient group.

## Discussion

PLE is a rare disorder characterized by the development of neuropsychiatric symptoms and associated with cancer in the absence of tumor cell invasion of the nervous system. PLE is often seen in SCLC, but can also be found in other cancers, such as breast cancer, thymoma, ovarian teratoma and Hodgkin lymphoma^8^. Our study assessed clinical characteristics, long-term prognoses, and anti-cancer treatment response rates in Asian patients with PLE who were subsequently diagnosed with lung cancer. Immune checkpoint inhibitors, such as nivolumab and ipilimumab, have become an important novel treatment for a variety of cancers recently. However, immune checkpoint blockade might trigger immune-related side effects, including LE. The course of most PLEs is subacute and symptoms occur before the detection of tumor. On the contrary, LE from immune checkpoint inhibitors emerges secondary to treatment, and usually responds well to drug withholding and rapid initiation of steroids^9,10^. In current study, no patients have received immune checkpoint inhibitors, obviating the need of differential diagnosis.

The pathologic features of PLE are believed to be immune-mediated, and include microscopic perivascular lymphocytic infiltration, neuronal cell loss, and reactive microglial proliferation of limbic structures^11^. Clinical manifestations typically include cognitive disorders, seizures, confusion, memory deficit, personality changes, and psychiatric symptoms. Nervous system involvement distant from the limbic system is frequently observed, including cerebellar ataxia, progressive encephalomyelopathy, peripheral neuropathy, and brain damage^1^. Thus, some cases in our study presented with gait and stance ataxia, static tremors, chronobiologic rhythm disturbances, and sensory polyneuropathy. Additionally, two patients had cerebral cortex hypometabolism as confirmed by PET-CT.

PLE diagnosis is generally based on a combination of clinical characteristics, and CSF, MRI, and EEG analyses. Additionally, metabolic encephalopathy, neurotoxic drugs, inflammatory disorders, central nervous system tumors, and neurodegenerative disorders must be excluded^12,13^. In our case series, MRI T2-FLAIR signals only confirmed bilateral temporal lobe hyperintensities in four patients, while PET-CT showed abnormal limbic system metabolism in nine LE cases. As far as we concerned, brain MRI is more useful to rule out other disorders, such as tumors, that might lead to neurologic symptoms. PET-CT might be more sensitive in the diagnosis and evaluation of PLE, especially in patients with normal MRI findings. Since nowadays, the diagnosis of PLE is still mainly based on clinical manifestations and antibody tests rather than imaging, further research is needed to evaluate the utility of PET-CT as a supplementary diagnostic method in PLE. In a study of GABA_B_ encephalitis by Kim, *et al*.^14^, three patients showed PET abnormalities, including one occurrence of cortical hypometabolism and two of temporal hypermetabolism. Cortical hypometabolism could be a characteristic of synaptic dysfunction, whereas mesiotemporal hypermetabolism might be related to inflammatory processes. Similarly, two GABA_B_ encephalitis patients in our study presented with cortical hypometabolism in PET-CT.

The identification of specific circulating autoantibodies in patients^5^, such as anti-Hu, anti-Yo, anti-Ma2, anti-amphiphysin, anti-NMDAR, anti-VGKC, and anti-GABA_B_R, has revolutionized the diagnosis of PLE and demonstrated immune system involvement. Onconeural antibodies, including anti-Hu, anti-amphiphysin, and anti-Yo, target antigens present in neuroectodermal tissues and tumors^15^. Since these antibodies target intracellular proteins and are predominantly associated with neuronal death and brain infiltration by cytotoxic T cells, patients expressing such antibodies are rarely sensitive to immunomodulatory treatment. On the contrary, humoral immunity plays a major role in patients with autoantibodies targeting cell surface antigens, and is associated with reversible neuronal dysfunction. Such patients achieve better recovery with prompt immunosuppressive treatment.

Half of the patients in our study had anti-GABA_B_R antibodies in serum or CSF. GABA_B_Rs are G-protein coupled receptors, and are typical inhibitory synaptic proteins in neurons, distributed in the hippocampus, thalamus, and cerebellum^16^. Anti-GABA_B_R encephalitis is a relatively rare disease, characterized in many cases by early and prominent seizure. Sixty to seventy percent of anti-GABA_B_R encephalitis patients show partial or complete response to immunotherapy and tumor therapy, since the disease is mediated by antibodies against cell surface antigens^7,17^. Our study confirmed this response rate. According to our results, all patients who achieved CR or PR from immunotherapy had antibodies against GABA_B_R. Additionally, the combination of steroids and IVIg yielded better responses than single agents alone. Anti-cancer treatment in the current study lead to better limbic symptom recovery outcomes than immunotherapy. Patients with anti-GABA_B_R-associated encephalitis, may also express additional autoantibodies^18^. The effects of these additional antibodies on anti-GABA_B_R encephalitis patient treatment response and prognosis require further investigation.

Immunomodulatory treatments, including corticosteroids, plasma exchange, immune adsorption, or IVIg, have been applied as first-line therapies in all types of LE^6,19^. A relatively small percentage of PLE patients experienced symptom stabilization or neurological improvement after immunomodulatory therapy, although most failed to achieve LE response until the primary tumor was controlled^1^. Surgery or systemic oncological treatment in the case of metastasis is recommended for LE patients with confirmed tumors^19^.

SCLC is sensitive to chemotherapy and radiotherapy. Concurrent platinum-etoposide and thoracic radiation therapy followed by prophylactic cranial irradiation with curative intent is standard for limited-stage SCLC, and platinum-etoposide remains the first-line therapy for extensive-stage disease^20,21^. Most PLE patients with SCLC experience improvement in neurological symptoms after systemic chemotherapy^22–24^. We found that neurological improvement was closely related to anti-cancer therapy (chemotherapy or VATS), rather than immunotherapy (Fig. 3A). Interestingly, neurological improvement in two patients was achieved after mediastinal lymph node resection via VATS. Therefore, we suggest that even slight reductions in tumor burden could lead to neurological improvement in PLE patients. Of note, patients with LE might have lower performance status scores owing to neurological disorders and some may require admittance into intensive care units due to status epilepticus^25^, which was previously considered contraindicative for chemotherapy. We recommend systemic assessments and/or multidisciplinary discussions before determining whether patients are suitable candidates for anti-cancer treatment. Aggressive chemotherapy, despite of low performance status score, might improve prognosis in this population.

Our study included one extensive-stage and 15 limited-stage lung cancer patients. Lung cancer patients with PLE might have more limited disease distributions and better long-term outcomes than those without PLE, due to immune-mediated eradication of tumor cells^26^. This hypothesis could explain why some patients in our study had isolated lymphadenopathy without an identifiable primary tumor. However, the overt neuropsychiatric symptoms of paraneoplastic syndrome prompted early screening and detection of the primary tumor. Therefore, the probability of delayed diagnosis and treatment might have been reduced for these patients, which is critical for better outcomes with rapidly progressing malignant tumors such as SCLC. In our study, 12/15 cases that received anti-cancer treatment survived through follow-up (Fig. 3B), and two-year overall survival reached 74.7%, which was much better than previously reported^7^. Six of eight patients with GABA_B_R antibodies achieved at least PR of their cancer after chemotherapy.

In conclusion, broad testing for anti-neuronal autoantibodies should be performed in patients with suspected LE, ideally in both CSF and serum to maximize detection rates. In patients with known lung cancer, especially SCLC, the development of LE without Hu antibodies is strongly associated with GABA_B_R antibodies. For PLE diagnosis, PET-CT could aid evaluations of both abnormal metabolism in the limbic system and the underlying neoplasm. Although some patients might benefit from immunosuppressive treatment, neurological symptoms only improve after tumor therapy in most cases. Since PLE is frequently seen in limited stage lung cancer, a strong suspicion for PLE might enable earlier diagnosis and lead to better long-term outcome.

## Materials and Methods

### Patients

We retrospectively reviewed the medical records of patients diagnosed with LE and lung cancer at Peking Union Medical College Hospital between January 2005 and January 2017. We identified 16 patients with LE and lung neoplasm. LE was diagnosed using the following criteria^1,27^: (1) short-term memory loss, seizures, or psychiatric symptoms suggesting limbic system involvement; (2) <4 years between onset of neurological symptoms and cancer diagnosis; (3) exclusion of metastasis, infection, metabolic and nutritional deficits, cerebrovascular disorder, or side effects of therapy that may cause limbic encephalopathy; and (4) at least one the following: CSF with inflammatory changes; MRI FLAIR or T2 presenting with unilateral or bilateral temporal lobe hyperintensities; and/or EEG showing slow- or sharp-wave activity in one or both temporal lobes. Lung cancer was diagnosed based on pathological confirmation, and histologic type was classified according to the World Health Organization’s histologic classification (2004). Lung cancer staging was based on the 2009 International Association for the Study of Lung Cancer Tumor Node Metastasis Staging Manual^28,29^.

Using standardized forms, two authors extracted medical information, including clinical presentation, serum and CSF anti-neuronal antibodies (anti-Hu, anti-Yo, anti-Ri, anti-GABA_B_R, anti-NMDAR, anti-AMPAR, anti-CASPR, anti-LGI, anti-amphiphysin, anti-CRMP5, anti-Ma2, anti-GM1), EEG, MRI characteristics, PET-CT, CSF analysis, and treatment type, along with outpatient follow-up information. Patients provided informed consent for participation in the current study at the time of diagnosis or follow-up. This study was approved by the Peking Union Medical College Hospital Institutional Review Board. Written informed consent to publish identifying images was obtained from all the patients.

### Statistical analysis

All statistical analyses were performed using SPSS 22.0 software (IBM-SPSS Inc., Chicago, IL, USA). Descriptive statistics were used to analyze clinical data, such as median and percentage. Continuous data were described using median and range. Survival curves were plotted using the Kaplan and Meier method.
